# A Bayesian Framework That Integrates Heterogeneous Data for Inferring Gene Regulatory Networks

**DOI:** 10.3389/fbioe.2014.00013

**Published:** 2014-05-20

**Authors:** Tapesh Santra

**Affiliations:** ^1^Systems Biology Ireland, University College Dublin, Dublin, Ireland

**Keywords:** network inference, Bayesian statistics, data interpretation, statistical, variable selection, gene regulatory networks

## Abstract

Reconstruction of gene regulatory networks (GRNs) from experimental data is a fundamental challenge in systems biology. A number of computational approaches have been developed to infer GRNs from mRNA expression profiles. However, expression profiles alone are proving to be insufficient for inferring GRN topologies with reasonable accuracy. Recently, it has been shown that integration of external data sources (such as gene and protein sequence information, gene ontology data, protein–protein interactions) with mRNA expression profiles may increase the reliability of the inference process. Here, I propose a new approach that incorporates transcription factor binding sites (TFBS) and physical protein interactions (PPI) among transcription factors (TFs) in a Bayesian variable selection (BVS) algorithm which can infer GRNs from mRNA expression profiles subjected to genetic perturbations. Using real experimental data, I show that the integration of TFBS and PPI data with mRNA expression profiles leads to significantly more accurate networks than those inferred from expression profiles alone. Additionally, the performance of the proposed algorithm is compared with a series of least absolute shrinkage and selection operator (LASSO) regression-based network inference methods that can also incorporate prior knowledge in the inference framework. The results of this comparison suggest that BVS can outperform LASSO regression-based method in some circumstances.

## Introduction

Understanding how genes regulate each other to orchestrate cellular phenotypes is a fundamental challenge of Biology. A straightforward way of uncovering gene regulatory networks (GRNs) is to perturb each gene of the network, e.g. by means of siRNAs and chemical inhibitors, and measure the effects of these perturbations on the expression of other genes in the network (Kholodenko et al., 2002; Wagner, 2002). However, the effects of such perturbations rapidly propagate through the entire network, causing widespread, global changes in the gene expressions, making it challenging to differentiate the direct interactions from the indirect ones. Several computational approaches were proposed to unmask the direct gene regulatory interactions by systematically analyzing perturbation responses (Kholodenko et al., 2002; Repsilber et al., 2002; Wagner, 2002; Gardner et al., 2003; Hartemink, 2005; Rogers and Girolami, 2005; de la Fuente and Makhecha, 2006; Margolin et al., 2006; Bansal et al., 2007). Many of these studies found that the steady-state perturbation responses of a gene are linearly dependent on the same of its direct regulators (Kholodenko et al., 2002; Gardner et al., 2003; Rogers and Girolami, 2005; de la Fuente and Makhecha, 2006; Bansal et al., 2007). These findings presented a unique opportunity of identifying direct genetic interactions by simply solving a set of linear equations. Although this approach seems simple in theory, implementing it in practice is not straightforward. First, biological measurements are noisy and contain experimental errors. The noise in biological datasets may cause significant errors while reconstructing GRNs by solving linear equations. Second, and perhaps most importantly, in order to solve these linear equations, one needs to perturb a GRN at least as many times as the number of genes in the network and measure the responses of all its genes after each perturbation (Kholodenko et al., 2002; Gardner et al., 2003; Rogers and Girolami, 2005; de la Fuente and Makhecha, 2006; Bansal et al., 2007). Therefore, reconstructing genome scale GRNs using the above method requires thousands (for simple organisms, e.g. bacteria, fungus, etc.) and often tens of thousands (for complex organisms such as mammals) of perturbation experiments that are time consuming and expensive. Most perturbation experiments, except those performed in some simple model organisms such as *Escherichia coli* (Baba et al., 2008) or yeast (Hughes et al., 2000), involve far fewer perturbations than the number of genes in the GRN. As a result, the datasets produced by these experiments do not have enough information for a full reconstruction (by solving linear equations) of the corresponding GRNs. Several statistical algorithms have been proposed to resolve this issue. For instance, some authors used singular value decomposition and linear regression (Yeung et al., 2002; Guthke et al., 2005; Zhang et al., 2010) to reconstruct GRNs using datasets obtained from a small number of perturbation experiments. Huang et al. (2010) used regulator filtering, forward selection, and linear regression for GRN reconstruction; and Imoto et al. (2003) used non-parametric regression embedded within a Bayesian network for the same purpose. Several other regression techniques such as the elastic net (Zou and Trevor, 2005; Friedman et al., 2010) and least absolute shrinkage and selection operator (LASSO; van Someren et al., 2003; Li and Yang, 2004; van Someren et al., 2006; Shimamura et al., 2007; Hecker et al., 2009, 2012; Lee et al., 2009; Charbonnier et al., 2010; Gustafsson and Hornquist, 2010; James et al., 2010; Pan et al., 2010; Peng et al., 2010; Wang et al., 2013) have also been widely used to reconstruct GRNs from noisy and insufficient perturbation response data.

Although many of these algorithms perform reasonably well, it is being increasingly clear that the accuracy of these algorithms can be significantly increased by integrating external data sources, e.g. gene sequence, single nucleotide polymorphism (SNP), protein–protein interaction (PPI), etc., in the network reconstruction process (Yeung et al., 2011; Lo et al., 2012). Public data repositories provide a rich resource of biological data related to gene regulation. Integrating data from these external data sources into network inference algorithms has become a primary focus of the systems and computational biology community. Previously, James et al. (2010) incorporated documented transcription factor binding sites (TFBS) information to infer the GRN of *E. coli*. Djebbari and Quackenbush (2008) used preliminary GRN derived from PubMed indexed literature and PPI databases as prior knowledge for their Bayesian network reconstruction algorithm. Zhu et al. (2004) combined TFBS and PPI data to infer GRNs. Imoto et al. (2003) used PPI, documented TFBS, and well studied pathways as prior information for their network inference method. Lee et al. (2009) presented a systematic way to incorporate various types of biological knowledge, such as the gene ontology (GO) annotations, data from ChIP–ChIP experiments, and a comprehensive collection of information about sequence polymorphisms. Yeung et al. (2005), Yeung et al. (2011), and Lo et al. (2012) developed a Bayesian model averaging approach to systematically integrate publicly available TFBS data, ChIP–ChIP data, physical interactions, genetic interactions, additional expression data, and literature curation.

This study is an extension of our previous work (Santra et al., 2013) which used a Bayesian framework that was designed to reconstruct biochemical networks by analyzing steady-state perturbation response data. In our previous study, we used Bayesian variable selection (BVS) algorithm to account for model uncertainty under noisy and insufficient data. Only generic topological knowledge such as sparsity of biochemical networks was used as prior information in the network reconstruction process. No external knowledge regarding potential interactions between network components was used to guide the inference process. The contributions of this study are four folds. First, a simple and an intuitive technique is proposed to incorporate external knowledge into the BVS framework in the form of a prior distribution. Second, a new way of integrating protein interactions among transcription factors (TFs) into the network inference framework is proposed. Although, PPI data were used previously (Zhu et al., 2008) in the context of GRN inference, the approach used by previous researchers was very different from the approach used in this study. For instance, protein interactions among target genes were used by Zhu et al. (2008) to determine co-regulation of multiple genes. Here, we use protein interaction among TFs to determine combinatorial regulations by multiple TFs. Third, as a proof of concept, the proposed methodology is applied to a gene expression dataset obtained from a liver-enriched TF regulatory network, revealing that it significantly outperforms our previous work. Finally, the performance of the proposed method is compared with a LASSO regression-based network inference method using publicly available gene expression datasets.

The rest of this study is organized as follows. In the next Section “Linear Model of Gene Regulation”, I briefly discuss linear models of gene regulation, followed by a detailed description of the proposed BVS algorithm in Sections “The Bayesian Variable Selection Algorithm” and “Sampling Scheme for the Proposed BVS Framework.” In Section “Integrating External Data to Formulate *P*(*A^i^*),” I present a new method of integrating external data sources in the BVS formulation. An implementation of this method to infer a liver-specific GRN is then discussed in Section “Inferring Liver-Specific Gene Regulatory Network from Perturbation Response Data.” In this section, I also compared the performance of the proposed BVS algorithm with our previous work. The results of comparing the proposed method with other network inference techniques are presented in Section “Inferring GRN of Human Breast Epithelium and Comparison with LASSO.” Finally, in the conclusion section, I discuss the advantages and disadvantages of our algorithm and future directions.

## Linear Model of Gene Regulation

When a GRN is perturbed, the effect of the perturbation rapidly propagates through the entire network, causing widespread, global changes in the expression levels of its genes. It has been shown (Rogers and Girolami, 2005; Bansal et al., 2007; Lo et al., 2012) that the responses (***x^i^*** = {x*_ij_, j* = 1, …, *n*_p_}) of a gene (*g_i_*), to a series of (*n*_p_) perturbations, are linearly dependent on the responses (***X^i^*** = {*x_kj_, k* = 1, …, *n_i_, j* = 1, …, *n*_p_, *k* ≠ *i*}) of its direct regulators (***g^i^*** = {*g_k_, k* = 1, …, *n_i_, k*≠*i*}), i.e.,

(1) $$x^{i}=X^{i^{T}}\beta^{i}$$

where *n_i_* is the number of regulators of the gene (*g_i_*), and ***β***^***i***^ = {β*_ik_, k* = 1, …, *n_i_, k* ≠ *i*} are the linear coefficients that represent the strengths and types of the interactions between the gene (*g_i_*) and its direct regulators (***g^i^***).

The measurements of the expression levels usually contain experimental errors, and may not exactly fit into the above Eq. 1. The difference between the left and right hand side of Eq. 1 caused by such errors are called the “residuals.” In order to compensate for errors, the residuals are added to Eq. 1 leading to,

(2) $$x^{i}=X^{i^{T}}\beta^{i}+\epsilon^{i}$$

where **ϵ**^***i***^ = {ϵ*_ij_, j* = 1, …, *n*_p_} represents the residuals caused by measurement errors. It can be easily showed that the residual variables (ϵ*_ij_*) are linear combinations of the individual measurement errors associated with the perturbation responses of the gene (*g_i_*) and its regulators (***g^i^***) (Kariya and Kurata, 2004). Since, the measurement errors are random in nature, the residual variables are also random variables, and by central limit theorem, these variables have Gaussian distribution (Kariya and Kurata, 2004). It is further assumed that the residual variables (**ϵ***^**i**^*) are independent of each other and have 0 mean and variance σ^2^ which depend on the extent of experimental/measurement error in the dataset (Rogers and Girolami, 2005; de la Fuente and Makhecha, 2006; Bansal et al., 2007; Vignes et al., 2011; Santra et al., 2013).

To identify the direct regulators (***g^i^***) of the gene (*g_i_*), one needs to calculate ***β^i^*** by solving Eq. 2 in a least-square sense. The elements (β*_ik_*) of ***β****^**i**^* whose absolute values are significantly >0 are then selected as direct interactions, and the corresponding genes (*g_k_*) are considered to be the direct regulators of *g_i_*. However, solving Eq. 2 requires at least as many perturbations as the number of genes (*n*) in the network (Kholodenko et al., 2002; Rogers and Girolami, 2005; de la Fuente and Makhecha, 2006; Santra et al., 2013). Under most circumstances, it is not possible to perform so many perturbation experiments, and therefore, in such cases, a full GRN reconstruction is not feasible by solving Eq. 2, either exactly or in a least-square sense. This issue is resolved by variable selection algorithms.

## Bayesian Variable Selection Algorithm

Variable selection algorithms find the most likely set of regulators (***g^i^***) for each gene (*g_i_*) by iteratively solving Eq. 2. It should be noted that the inferred interactions between a gene (*g_i_*) and its regulators (***g^i^***) may not always represent causal relationships. In many cases, these interactions represent “acausal” dependencies between gene expressions (Guyon and Elisseeff, 2003). Yet, it has been shown that variable selection algorithms can infer gene regulatory programs with reasonable accuracy (Yeung et al., 2005, 2011; Lo et al., 2012). The mechanism of a simple variable selection technique in the context of GRN reconstruction is described below.

(a) First, a random set of genes $\left(g_{1}^{i}\right)$ are selected as the potential regulators of a gene (*g_i_*), and the least-square estimates $\left(\beta_{1}^{i}\right)$ of the corresponding interaction strengths and the resulting sum of square error $\left(\epsilon_{i1}^{\text{SOS}}=||\epsilon_{1}^{i}|{|}^{2}\right)$ are calculated.
(b) At the next iteration, a different set of genes $\left(g_{2}^{i}\right)$ are selected as the potential direct regulators of gene *g_i_*, and again, the least-square estimates $\left(\beta_{2}^{i}\right)$ of corresponding interaction strengths and the resulting sum of square error $\left(\epsilon_{i2}^{\text{SOS}}=||\epsilon_{2}^{i}|{|}^{2}\right)$ are calculated.
(c) The newly calculated sum of square error $\left(\epsilon_{i2}^{\text{SOS}}\right)$ is then compared with the one $\left(\epsilon_{i1}^{\text{SOS}}\right)$ calculated in the previous iteration. If $\epsilon_{i2}^{\text{SOS}}<\epsilon_{i1}^{\text{SOS}}$, then the new set of potential regulators $\left(g_{2}^{i}\right)$ is considered more likely to directly regulate *g_i_* than the previous one $\left(g_{1}^{i}\right)$, otherwise the old set is retained as the most likely potential regulators.
(d) For each gene (*g_i_*), the above procedure is repeated for all possible combination of potential regulators until a set of regulators is found that has the minimum sum of squared error.

The above scheme is simple in theory, but there are some major obstacles in implementing it in practice. For instance, if we want to reconstruct a GRN involving 1000 genes, then, for each gene, we need to iterate through 2^999^ possible combinations of potential regulators to find its most likely direct regulators. Iterating through so many possible combinations is not feasible even for the most advanced computing systems. Therefore, we must adopt a smarter strategy to find the most likely set of regulators of each gene in a GRN. BVS algorithms (in general) implement efficient search strategies to identify the most likely regulators of a gene in a reasonable time. Here, I adopted a BVS framework which is similar to our previous work (Santra et al., 2013) with a few exceptions.

To formulate the BVS algorithm, it is convenient to represent the topology of a GRN using a binary “adjacency” matrix (***A***). A non-zero entry (*A_ik_* = 1, *k* ≠ *i*) of this matrix represents direct regulation of one gene (*g_i_*) by another (*g_k_, k*≠*i*), whereas the zero elements indicate no direct regulation. Consequently, the non-zero elements of the *i*th row (***A^i^*** = {*A_ik_, k* = 1, …, *n, k* ≠ *i*}) of this matrix represent interactions between the gene *g_i_* and its direct regulators (***g^i^***). Note that the binary adjacency matrix (***A***) and the matrix of interaction strengths (***β***) are closely related, since absence of direct interaction (*A_ik_* = 0, *i* ≠ *k*) between two genes (*g_i_, g_k_*) implies zero interaction strength (β*_ik_* = 0, *i* ≠ *k*). In other words, the elements (β*_ik_, i* ≠ *k*) of the interaction strength matrix (***β***) corresponding to the zero elements (*A_ik_* = 0, *i* ≠ *k*) of the binary adjacency matrix (***A***) are also zero. Therefore, finding the most likely direct regulators of a gene (*g_i_*) amounts to finding the most likely combination of 0s and 1s in the *i*th row (***A^i^***) of the binary matrix ***A***.

To avoid iterating through all possible combinations of ***A^i^***, BVS algorithms adopt a Bayesian approach. Bayesian algorithms closely mimic the natural learning process of human brain that updates its knowledge about certain events when it receives new information about the event. In these algorithms, the prior knowledge about a certain event is represented by its prior distribution which assigns a prior probability to each possible outcome of the event. When new information becomes available, the prior probabilities are updated using Bayes’ theorem. The updated probability distribution is known as the posterior distribution. The posterior distributions represent our up-to-date knowledge about a certain event based on the data that have been recently available.

In the context of GRN reconstruction, any prior knowledge about the possible regulators (***g^i^***) of each gene (*g_i_*) is encoded in the prior distributions (*P*(***A^i^***)) of the binary vectors ***A^i^***. In our previous work (Santra et al., 2013), we formulated the prior distribution *P*(***A^i^***) to penalize gene regulation models with too many regulators and favored sparse models where each gene is regulated by a small number of regulators. No other external knowledge was used to formulate the prior distribution of ***A^i^***. Here, we take a different approach and formulate a more informative prior distribution of ***A^i^*** by integrating TFBS and PPI between TFs. The process of integrating PPI and TFBS data into the prior distribution of ***A^i^*** is an important aspect of data integration and will be discussed in detail in the next section.

Prior information about the possible values of the interaction strengths (***β***^***i***^) is rarely available. In the absence of any specific prior knowledge of the possible values of ***β***^***i***^, it is safe to assume that its non-zero elements can take a wide range of positive or negative values depending on whether the corresponding interaction is activating or repressing. The zero elements represent no direct interaction and correspond to the zero elements of ***A^i^***. This assumption is formulated by assigning a multivariate Gaussian prior to the non-zero elements of ***β***^***i***^. The prior distribution of ***β***^***i***^ is assumed to have zero mean and covariance matrix $V_{\beta^{i}}$, which is a (*n_i_* × *n*_i_) matrix that represents our prior knowledge about the possible ranges of values of ***β***^***i***^. A common approach is to assume that the prior covariance matrix ($V_{\beta^{i}}$) of ***β***^***i***^ is proportional to the scaled fisher information matrix (FIM) of ***β***^***i***^, i.e. $V_{\beta^{i}}=c\sigma^{2}{\left(X^{i^{T}}X^{i}\right)}^{-1}$, where *c* is the proportionality constant (also known as Zellner’s constant) which determines the span of the prior distribution of ***β***^i^ (Zellner, 1986; Ishwaran and Rao, 2005; Gupta and Ibrahim, 2009) and σ^2^ is the scaling factor which is the same as the variances of the residual variables ϵ*_ij_*. The above formulation of the covariance matrix assumes that the variances/covariances of the interaction strengths depend not only on the inherent variability of the perturbation responses, but also on the variance of the measurement errors. It was shown by other researchers that the choice of the proportionality constant *c* has a significant impact on the performance of BVS algorithms and several studies were conducted to find the most appropriate value of *c* (George and Foster, 2000; Fernández et al., 2001; Hansen and Yu, 2001; Liang et al., 2008). Fernández et al. (2001) demonstrated that among the commonly used values, $c=\max(n_{p},n_{i}^{2})$ performs the best in most scenarios. Therefore, this value was chosen for the BVS framework presented in this study.

The prior knowledge about the noise variance σ^2^ is incorporated in its prior distribution. Previously, the noise variance σ^2^ was assumed to have a gamma distribution with shape and scale parameters, α and β, respectively (Santra et al., 2013). The values of these parameters were set to 1 to ensure a flat prior, which represents our lack of prior knowledge about extent of noise in the dataset. Here, in order to avoid extra hyper parameters (α, β), we assumed that σ^2^ has Jeffrey’s prior (Fernández et al., 2001), i.e. $p\left(\sigma^{2}\right)∼\frac{1}{\sigma^{2}}$, which is an uninformative “improper” prior that relies on the notion that noises in biological data are unlikely to cause very large residuals in the linear models.

These prior distributions can then be updated to posterior distributions based on the measured perturbation responses of the network using Bayes formula. Here, we are interested in the posterior distributions of binary vectors ***A^i^**, i* = 1, …, *n*, since these vectors represent the network topology. It is straightforward to show that the posterior distribution (*P*(***A^i^***|***x^i^**, **X^i^***)) of ***A^i^*** given the perturbation responses of gene *g_i_* and its regulators is (Liang et al., 2008; Note 1 in Supplementary Material)

(3) $$P\left({\text{A}}^{\text{i}}|{\text{x}}^{\text{i}},{\text{X}}^{\text{i}}\right)\propto \left[{\left(1+c\right)}^{-\left(\frac{n_{i}+1}{2}\right)}{\left(1-\frac{c}{1+c}R^{2}\right)}^{\frac{-\left(n_{p}-1\right)}{2}}\right]P\left({\text{A}}^{\text{i}}\right)$$

here $R^{2}=1-\frac{{\left(x^{i}-X^{i^{T}}\hat{\beta^{i}}\right)}^{T}\left(x^{i}-X^{i^{T}}\hat{\beta^{i}}\right)}{{\left(x^{i}-\bar{x^{i}}\right)}^{T}\left(x^{i}-\bar{x^{i}}\right)}$ is the coefficient of determination of the linear model shown in Eq. 2, where β^i=XiTXi−1XiTxi is the least-square estimate of ***β***^***i***^, and ${\bar{x}}^{i}$ is the sample average of ***x^i^***.

Finding the most likely regulators of gene *g_i_* is equivalent to finding the configuration of ***A^i^*** that maximizes the above posterior probability (Eq. 3). But, as discussed before, finding this configuration requires iterating through all possible configurations of ***A^i^***, which is hardly possible for large networks. An alternative approach is to estimate the “expected” configuration of ***A^i^*** using model averaging techniques that identify a number of “good enough” configurations instead of a single “best” configuration. The average of these good configurations is commonly used as an approximation of the “expected” configuration of ***A^i^***. The “good enough” configurations of ***A^i^*** can be determined in reasonable time by drawing samples from the above posterior distribution (Eq. 3) using a Markov Chain Monte Carlo (MCMC)-based sampling algorithm.

## Sampling Scheme for the Proposed BVS Framework

A typical MCMC-based sampling algorithm iteratively explores different configurations of ***A^i^*** in order to find those with relatively high posterior probability. In each iteration, it calculates the posterior probability of the current and a proposed new configuration of ***A^i^***. However, in some cases, it is not possible to calculate the posterior probability of certain configurations of ***A^i^***. For instance, when $n_{i}\gg n_{p}$, i.e. the number of 1s in ***A^i^*** is larger than the number of perturbations, then the corresponding data matrix ***X^i^*** has dimensions *n*_p_ × *n*_i_ and suffers from rank deficiency. Therefore, the Gram matrix $X^{i^{T}}X^{i}$ is non-invertible and the corresponding coefficient of determination (***R***) and the posterior probability (*P*(***A^i^***|***x^i^**, **X^i^***)) do not exist. Previously (Santra et al., 2013), we addressed this issue by adding a diagonal loading ($X^{i^{T}}X^{i}+\delta I$) to the Gram matrix, ensuring its invertibility. However, this approach requires the estimation of an optimal value for the loading constant (δ), which adds to the complexity of the sampling process. Additionally, the effects of diagonal loading on the overall outcome of BVS algorithms are not well understood. In this study, a different strategy is adopted to address the above issue. Here, in order to avoid rank deficiency, the search space (ζ) of the MCMC algorithm is constrained to only those configurations of ***A^i^*** which has less number of 1s than the number of perturbations, i.e. *n_i_* < *n*_p_. The restricted search space is denoted by $\zeta_{n_{\text{p}}}$ $\left(\zeta_{n_{\text{p}}}\subset \zeta \right)$, where the subscript *n*_p_ indicates the upper limit on the number of 1s in the configurations of ***A^i^***. The above approach has two major advantages over the previous method. First, it ensures the existence of the posterior probability without artificial diagonal loading of the Gram matrix. Second, it decreases the computational complexity of the MCMC algorithm by reducing the size of the data matrix ***X^i^***. This property makes this approach particularly attractive for inferring large GRNs where computational complexity is a major issue for MCMC-based variable selection algorithms. The computational cost of sampling can be significantly reduced by further restricting the search space to an even smaller subspace $\left(\zeta_{k}\subseteq \zeta_{n_{\text{p}}}\right)$, which contains only those configurations of ***A^i^*** that have less than *k* (where *k* ≤ *n*_p_) numbers of 1s. Restricting the search space to ζ*_k_* implies that the MCMC algorithm will explore regulatory programs (configurations of ***A^i^***) consisting of at most *k* ≤ *n*_p_ regulators for each gene (*g_i_*). For accurate network inference, it is therefore desirable to assign the restriction parameter (*k*) a reasonable value that is not far from the ground truth. Although, there is no easy way of determining an optimal *k*, one can use prior information about the topology of the network to have a broad estimate of this parameter. This is discussed in the results section where the proposed algorithm is implemented on experimental data sets to infer GRNs. In the rest of this section, I continue with the discussion of the MCMC-based sampling algorithm, which is used in this study to explore the restricted search space (ζ*_k_*) of potential gene regulatory programs.

A Metropolis–Hastings algorithm was implemented to systematically explore ζ*_k_* and identify highly probable regulatory programs (***A^i^***). The sampling algorithm starts with a random configuration of ***A^i^*** ∈ ζ*_k_*. A new configuration $A^{i\prime }\in \zeta_{k}$ is then proposed based on a proposal distribution *Q*. The proposal distribution (*Q*) is formulated as follows. Let η(***A^i^***) ⊆ ζ*_k_* denote a set of binary vectors consisting of all possible configurations that can be obtained by changing one of the elements of ***A^i^*** from 0 to 1 or vice versa. Define a proposal distribution *Q* as follows.

(4) $$Q\left({\text{A}}^{\text{i}},{\text{A}}^{\text{i}\prime }\right)=\left\{\begin{array}{cc} =\frac{1}{\left|\eta \left({\text{A}}^{\text{i}}\right)\right|}\; & if{\text{A}}^{\text{i}\prime }\in \eta \left({\text{A}}^{\text{i}}\right)\\ 0\; & if{\text{A}}^{\text{i}\prime }\notin \eta \left({\text{A}}^{\text{i}}\right)\\ \; \end{array}\right.$$

Based on the above proposal distribution, an acceptance ratio $\alpha =\frac{P\left(A^{i\prime }|X^{i\prime },x^{i}\right)Q\left(A^{i\prime },A^{i}\right)}{P\left(A^{i}|X^{i},x^{i}\right)Q\left(A^{i},A^{i\prime }\right)}$ is computed. The proposed new configuration A^i′^ is then accepted with probability min(1, α). If accepted, A^i′^ is added to the sequence of drawn samples and becomes the current configuration. Else, ***A^i^*** remains the current configuration. Repeating this procedure in an iterative manner gives rise to an irreducible Markov chain in the restricted search space (ζ*_k_*). This Markov chain asymptotically converges (Geyer, 2011) to the desired posterior (*P*(***A^i^***|***x^i^**, **X^i^***)). Upon convergence, the samples drawn by the chain resemble those drawn from the posterior (*P*(***A^i^***|***x^i^**, **X^i^***)), and therefore, the most probable configurations of ***A^i^*** appear more frequently in the drawn samples than the improbable ones. These samples are then used to determine an “average” or “expected” regulatory program for each gene (*g_i_*). The expected probability that a gene (*g_i_*) is regulated by another gene (*g_k_*) is estimated by calculating the ratio of the number (*n_ij_*) of samples whose *j*th element is 1 to the total number (*n_s_*) of samples, i.e. $P\left(A_{ij}=1|x_{i},X^{i}\right)=\frac{n_{ij}}{n_{s}}$ (Mukherjee and Speed, 2008). Calculating this probability for each pair of genes results in a probabilistic representation of the network topology.

The above sampling algorithm draws samples from the posterior distribution of ***A^i^*** (Eq. 3) which depends on its prior distribution. This can be exploited to incorporate prior knowledge from external data sources into the BVS algorithm. To do so, the prior distribution (P(***A^i^***)) needs to be formulated in such a way that it favors the likely interactions supported by external data sources. This will bias the posterior of ***A^i^*** toward the interactions that are supported by external data. Below, I show a scheme that integrates TFBS with PPI information to formulate the prior distribution *P*(***A^i^***).

## Integrating External Data to Formulate *P*(*A^i^*)

Genes regulate each other via several mechanisms, e.g. transcriptional regulation, methylation, histone acetylation, etc. Among the known mechanisms of gene regulation, transcriptional regulation via TFs is perhaps the most well-studied gene regulatory mechanism. In the case of transcriptional regulation, proteins produced by regulatory genes undergo post-translational modifications and then either directly bind to the promoter regions of target genes or form multi-protein transcription factor complexes (TFCs) that bind to the gene promoters and regulate the activity of the corresponding genes. The regulatory proteins and TFCs bind genes at specific locations containing specific nucleotide sequences, commonly referred to as TFBS. These binding sites are experimentally determined by ChIP–ChIP experiments (Hughes et al., 2000) and/or computationally predicted by statistical algorithms (Matys et al., 2006; Bryne et al., 2008; Bailey et al., 2009; Ernst et al., 2010). There are a number of databases that contains vast amount of information on binding specificities (TFBS) of several TFs and TFCs (Matys et al., 2006; Bryne et al., 2008; Bailey et al., 2009). However, there are some limitations of incorporating these informations as prior knowledge into a network inference algorithm. First, the binding specificities are known only for a fraction of all TFs and TFCs that are found in nature. For a large number of TFs and TFCs, such information is unavailable. It is challenging to interpret the unavailability of information in an unambiguous manner. For instance, it is difficult to determine whether the lack of information represents absence of interaction or simply lack of knowledge about the presence of interaction. Second, TFs may indirectly regulate genes by forming protein complexes (TFCs) with other TFs which directly bind to gene promoters. Many of these indirect regulations are not well characterized, further contributing to the incompleteness of prior knowledge regarding gene regulation.

To address the above issues, I propose a simple scheme of incorporating available knowledge into the prior distribution of ***A^i^***. The proposed prior distribution favors potential regulatory interactions supported by TFBS data available in public databases. However, it does not exclude the possibility of potentially new interactions that are not supported by external sources. Furthermore, it uses information regarding protein interactions among the TFs to determine potential indirect gene regulations. These indirect regulatory interactions, along with the TFBS specificities, are then collectively used as potential regulatory interactions in the formulation of the prior distribution of ***A^i^***. A step-by-step description of using external data sources to formulate the prior distribution of ***A^i^*** is shown below.

**Figure 1:**
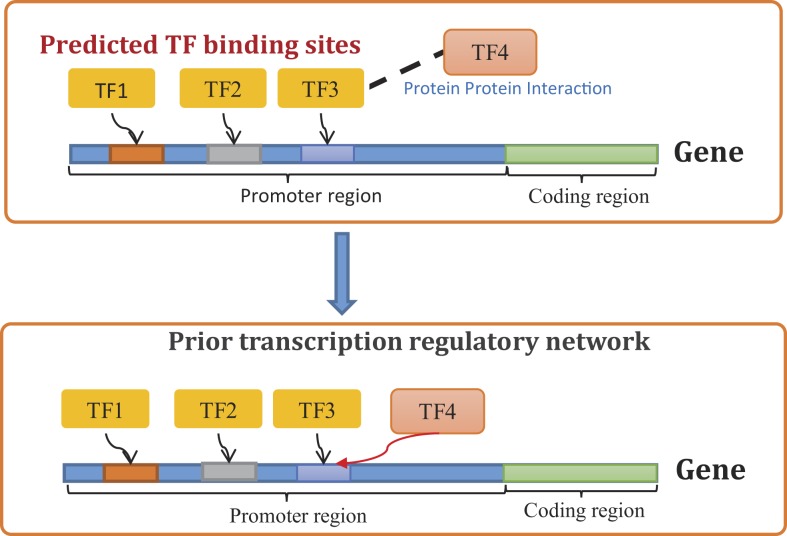
**Constructing prior transcription regulatory network using TFBS and PPI data**.

*Step 1*: First, TFBS information are collected from multiple external sources, e.g. public databases such as HTRIDB (Bovolenta et al., 2012), ENCODE (Hughes et al., 2000), KEGG (Ogata et al., 1999), ConsensusPathDB (Kamburov et al., 2011), etc., published literature (Ernst et al., 2010), computational TFBS prediction services such as MEME (Bailey et al., 2009), TRANSFAC (Bryne et al., 2008), JASPER (Matys et al., 2006), TRED (Jiang et al., 2007), etc.
*Step 2*: Next, information regarding PPIs among known TFs are obtained from publicly available sources. Recently, Ravasi et al. (2010) determined a comprehensive map of physical interactions among mammalian TFs using mammalian two-hybrid system. They identified around 800 protein interactions among human and mouse TFs. Arguably, this dataset is the most reliable source of information regarding protein interactions among TFs and is used in the large-scale GRN inference study later in this study. However, Ravasi et al.’s study does not cover all mammalian TFs, in which case proteins interaction databases such as STRING (Szklarczyk et al., 2011), HPRD (Keshava Prasad et al., 2009), IntAct (Kerrien et al., 2012), BIND (Bader et al., 2003), KEGG (Ogata et al., 1999) is used to determine PPI between TFs. It should be noted that many of these databases store functional and computationally predicted PPIs which may not always represent physical protein bindings. Since, we are interested in physical interactions among TFs, only physical PPIs are carefully selected from the above databases, functional and computationally predicted PPIs are excluded from the list of potential TF–TF protein interactions.
*Step 3*: The above information is then used to build a prior network that contains both direct and indirect regulations supported by external data. Potential direct regulations are identified using TFBS information as described above (see Step 1). Potential indirect regulations are identified based on the assumption that if a TF binds to another TF which targets a certain gene, then the former indirectly regulates the target of the later (Figure 1). Both direct and indirect regulations are incorporated in the prior network as potential transcriptional interactions. The prior network is represented by a weighted adjacency matrix (**Γ**). The non-zero elements of this matrix represent potential transcriptional regulations supported by TFBS and PPI data. The value of a non-zero element (Γ*_ij_* ≠ 0) represents our confidence on the regulation of a gene (*g_i_*) by another (*g_j_*). In this study, equal confidence is placed on all potential transcriptional regulations that are supported by TFBS and PPI data, i.e., Γ*_ij_* = α_c_ if gene *g_i_* has a TFBS for *g_j_* or any of its binding partners. Here, α_c_ is called the confidence parameter. The *i*th row (**Γ**^***i***^) of the prior adjacency matrix (**Γ**) represents our prior knowledge about the regulatory program of gene *g_i_* and is used to formulate the prior distribution of the binary vector ***A^i^*** in the following manner.
  (5) $$\begin{array}{llll} & P\left({\text{A}}^{\text{i}}\right)\propto \exp\left(\Gamma^{{\text{i}}^{\text{T}}}{\text{A}}^{\text{i}}\right):{\text{A}}^{\text{i}}\in \zeta_{k}\; & & \;\\ & =0\text{ otherwise}. \end{array}$$

The above prior distribution ensures that the prior probability of ***A^i^*** ∈ ζ*_k_* depends only on the number of interactions (*A_ij_* = 1) which are supported by prior information (Γ*_ij_* = α_c_). This implies that if two different configurations of ***A^i^*** have different numbers of potentially new interactions (*A_ij_* = 1, Γ*_ij_* = 0) but the same number of previously known interactions (*A_ij_* = 1, Γ*_ij_* = α_c_), then these two configurations have the same prior probability. Therefore, the above prior distribution (Eq. 5) favors regulatory programs (configurations of ***A^i^***) that have large number of known interactions (Γ*_ij_* = α_c_) but does not penalize the presence of previously unknown interactions, allowing such interactions to be seamlessly inferred by the variable selection algorithm.

As a proof of concept, I implemented the above BVS algorithm to reconstruct a liver-specific transcription regulatory network by analyzing perturbation response data. To show the effectiveness of integrating TFBS and PPI data in the BVS framework, I used four different prior settings for ***A^i^***. In the first setting, no external data source was used to formulate the prior distribution of ***A^i^*** and all possible regulatory programs (configurations of ***A^i^***) were considered equally likely *a priori*. In the second setting, no external data sources were used, but the prior distribution of ***A^i^*** was designed to favor sparse regulatory programs, i.e., the configurations of ***A^i^*** which has relatively fewer non-zero elements than zero elements. This approach is similar to that we adopted in our previous work (Santra et al., 2013). In the third setting, a prior network was constructed using only direct regulatory interactions that were predicted from publicly available TFBS information. This prior network was then used to formulate the prior distribution of ***A^i^*** as shown in Eq. 5. In the final setting, I used both direct and indirect regulatory interactions that were predicted from both TFBS and PPI interaction data to construct the prior network. This prior network was then used to formulate the prior distribution of ***A^i^***. The results of the above analysis are described in detail in the following section.

## Inferring Liver-Specific Gene Regulatory Network from Perturbation Response Data

Genes that play key roles in liver development, physiology, and disease are found to be tightly regulated by a handful of TFs, such as hepatocyte nuclear factors (HNF1A, HNF1B, HNF3A, HNF3B, HNF3G, HNF4A, HNF4G, and ONECUT1), CCAAT/enhancer-binding proteins (CEBPA and CEBPB), peroxisome proliferator activated receptors (PPARA, PPARD, and PPARG), retinoic acid receptors (RARA, RARB, and RARG), retinoid receptors (RXRA, RXRB, and RXRG), and RAR-related orphan receptors (RORA and RORC) (Schrem et al., 2002, 2004; Odom et al., 2004, 2006; Tomaru et al., 2009). The genes that encode these TFs are known to transcriptionally regulate each other to maintain a particular sequence of events leading to the normal development of liver tissues (Schrem et al., 2002, 2004; Odom et al., 2004, 2006; Tomaru et al., 2009). Therefore, uncovering the GRN involving the above genes is a fundamental step in understanding the physiological processes of liver development. For this purpose, Tomaru et al. (2009) perturbed the above GRN, one gene at a time, using siRNAs and measured the steady-state expression levels of these genes after each perturbation. Here, these measurements were used to infer the topology of the above GRN.

As mentioned above, four different versions of the aforementioned BVS framework were used for network inference, each with a different prior distribution of ***A^i^***. In the first case, all configurations of ***A^i^*** were assumed to have equal prior probability, i.e. *P*(***A^i^***) = γ, where γ is a constant.

In the second case, the prior distribution of ***A^i^*** was designed to assign higher probabilities to those configurations of ***A^i^*** which have fewer ones than zeroes. For this purpose, ***A^i^*** was assumed to have a beta binomial distribution,

(6) $$P\left({\text{A}}^{\text{i}}\right)=\left(\begin{array}{c} n_{\text{r}}\\ n_{i} \end{array}\right)\frac{B\left(n_{i}+\alpha ,n_{\text{r}}-n_{i}+\beta \right)}{B\left(\alpha ,\beta \right)}$$

Here, *n*_r_ is the number of potential regulators in gene *g_i_*. When all genes in the network are considered to be the potential regulators of *g_i_, n*_r_ = *n* − 1. The values of the shape parameters (α, β) were kept the same as those used in our previous work (Santra et al., 2013), i.e. α = 1, β = 2.

**Figure 2:**
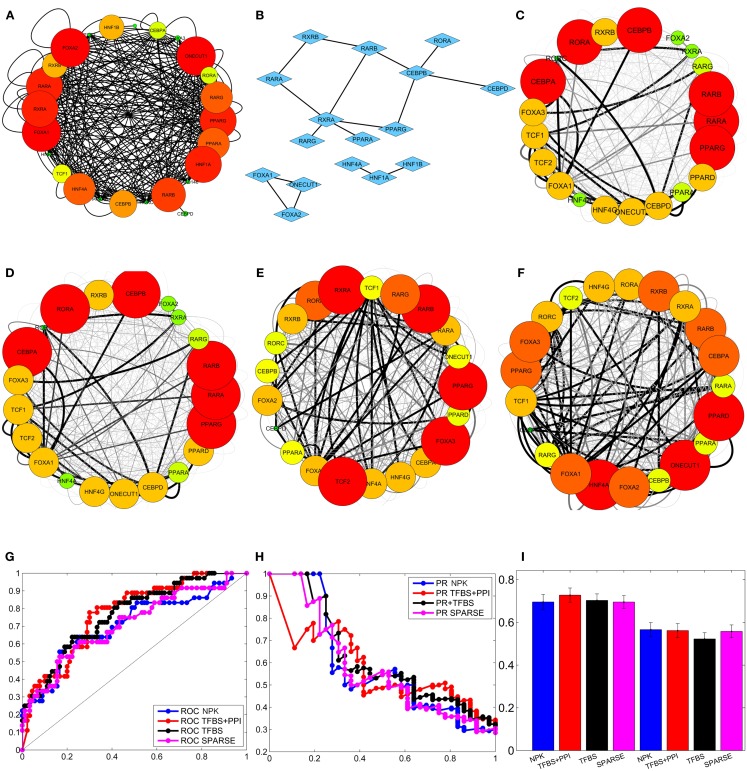
**Integrating external data to infer liver-specific transcription regulatory network (Tomaru et al., 2009)**. **(A)** Prior network constructed from TFBS information. **(B)** PPI among transcription factors. **(C)** Network inferred by flat prior (*P*(***A^i^***) = γ)). **(D)** Network inferred using sparse prior. **(E)** Network inferred using prior network constructed from TFBS information only. **(F)** Network inferred using prior network constructed from TFBS and PPI information. The interactions that occur with high and low posterior probabilities are represented by darker/thicker and lighter/thinner edges, respectively, in **(C)**, **(D)**, **(E)**, and **(F)**. **(G)** Average ROC curves of the inferred networks. **(H)** Average PR curves of the inferred networks. **(I)** mean and standard deviation of the area under the ROC and PR curves of the inferred networks.

In the third setting, only TFBS information was used to construct the prior network (Figure 2A). TFBS information were collected from HTRIDB (Bovolenta et al., 2012), MEME (Bailey et al., 2009), TRANSFAC (Bryne et al., 2008), JASPER (Matys et al., 2006), TRED (Jiang et al., 2007), and SABioscience (www.sabiosciences.com). Here, only those TFBS that were found within a 5000 bp region of the gene promoters were included in the analysis. This resulted in a total of 106 potential transcriptional regulations (excluding autoregulations, see Table S1 in Supplementary Material for details) among the 21 TFs mentioned above. These regulatory interactions were represented by a prior adjacency matrix (**Γ**_**TFBS**_) whose non-zero elements represent potential gene regulations and are assigned a value of α_c_ = 2. The *i*th row $\left(\Gamma_{TFBS}^{i}\right)$ of this matrix (**Γ**_**TFBS**_) represents our prior knowledge on the regulatory program of the *i*th gene *g_i_*, based solely on TFBS information, and was used to formulate the prior distribution of ***A^i^***.

In the fourth setting, both TFBS and PPI among TFs (Figure 2B; Table S2 in Supplementary Material) were used to determine potential gene regulations. The TFBS information was collected as described above. Information regarding PPIs among the above TFs was obtained from STRING (Szklarczyk et al., 2011) and HPRD (Keshava Prasad et al., 2009) databases (Table S2 in Supplementary Material). The TFBS and PPIs were used to determine potential direct and indirect regulatory interactions as described in the previous section (see Figure 1). These resulted in a total of 217 potential gene regulatory interactions (excluding autoregulations; see Table S3 in Supplementary Material for details) which were used to construct the prior network matrix (**Γ**_**TFBS+PPI**_). The non-zero elements of this matrix (**Γ**_**TFBS+PPI**_) were assigned a value of α_c_ = 2. The rows of the prior matrix (**Γ**_**TFBS+PPI**_) were then used to formulate the prior distributions *P*(***A^i^***), *i* = 1, …, *n*.

In all the above cases, the search space for the MCMC sampler was restricted to ζ*_k_*, where the subscript *k* represents the upper limit on the number of regulators for each gene. The value of *k* was chosen to be the same as the average number of regulators per gene $\left(\frac{217}{21}\approx 10\right)$ in the prior network (**Γ**_**TFBS+PPI**_) constructed from TFBS and PPI data.

The GRNs reconstructed using the above prior settings were then compared to a gold standard network (GSN) which was deduced by Tomaru et al. (2009) using matrix RNAi combined with rt-qPCR and Chromatin Immunoprecipitation (X-ChIP) experiments (see Figure S1 in Supplementary Material). To reconstruct the GSN, Tomura et al. knocked down 19 of the above genes, one at a time, and measured the responses of these genes to each knockdown. If a gene responded to the knockdown of another, then the former was considered to be potentially regulated by the later. Based on this assumption, a set of potential gene regulatory interactions (***G***_**RNAi**_) were determined. This was followed by X-ChIP/qPCR analysis that determined the DNA binding preferences of six (TCF1, FOXA1, FOXA2, HNF4A, ONECUT1, and RXRA) of the above TFs. If a TF was found on the promoter of a target gene in the X-ChIP experiment, then the later was considered to be potentially regulated by the former. A second set of potential gene regulations (***G***_**XChIP**_) were identified based on the X-ChIP measurements. The set of interactions (***G***_**ref**_) that were common to both the above networks (***G***_**RNAi**_ and ***G***_**XChIP**_) were then considered to represent the GSN (***G***_**ref**_ = ***G***_**RNAi**_ ∩ ***G***_**XChIP**_). The networks inferred by the proposed BVS frameworks with different prior setting were then compared with the above GSN. Since the GSN contains information regarding the regulatory activities of only six (out of 21) TFs, I compared only the interactions involving these TFs. The activities of the remaining 15 TFs were excluded from the comparison.

Recall that the proposed BVS algorithm uses MCMC sampling to estimate the posterior interaction probabilities. These posterior probabilities represent the *a posteriori* confidence on each interaction based on the perturbation response, TFBS and PPI data. If the posterior probability of an interaction is higher than a certain threshold (*p*_τh_), then the corresponding interaction is considered to be a true interaction. On the other hand, if a posterior probability is less than or equal to this threshold, then the corresponding interaction is thought to be absent in the GRN. This implies that the topology of the reconstructed GRN depends on the threshold probability (*p*_τh_) and therefore, any comparison between the reconstructed GRN and the true GRN also depends on the choice of this threshold. For a more objective assessment, multiple GRNs are constructed from the above posterior probabilities using multiple different thresholds. Each reconstructed GRN is then compared with the true GRN and the number of correctly and incorrectly inferred interactions are counted. These counts are used to calculate the true positive rates (TPRs), false positive rates (FPRs), and precisions (PREs) of the reconstructed GRNs. The TPR is the ratio of total number of the correctly identified interactions to the total number of interactions present in the GSN (Fawcett, 2004; Powers, 2011); the FPR is the ratio of the total number of incorrectly identified interactions and the total number of possible interactions that are absent in the GSN (Fawcett, 2004; Powers, 2011); PRE is the ratio of the total number of correctly identified interactions to the total number of interactions present in the inferred network. Then, the TPRs (*Y*-axis) are plotted against the FPRs (*X*-axis), and the PREs (*Y*-axis) are plotted against TPRs (*X*-axis) in two separate plots, commonly known as receiver operating characteristic (ROC) and precision recall (PR) curves, respectively (Fawcett, 2004; Powers, 2011). The areas under these curves, denoted by AUROC and AUPR, give an objective assessment of the accuracy of the GRNs reconstructed by the BVS algorithms (Fawcett, 2004; Powers, 2011). Both AUROC and AUPR can have values between 0 and 1, and the closer these values are to 1, the better is the accuracy of the inferred networks, with AUROC = 1 and AUPR = 1 being the ideal case. To perform a robust comparison, the proposed BVS algorithm was executed 50 times under each prior setting, producing 50 posterior networks for each prior network (see Figures 2C–F for sample posterior networks inferred from different priors). ROC, PR curves, and the areas under these curves (AUROC and AUPR, respectively) were calculated from each posterior network. The average ROC and PR curves of the networks that were inferred from the same network prior was then calculated for each prior setting (Figures 2G,H). The mean and standard deviations of the corresponding AUROC and AUPR values, calculated under different prior settings, are shown in Figure 2I. The AUROC values calculated under different prior settings were then compared using Mann–Whitney *U* test (Mann and Whitney, 1947) to assess the effects of different network priors on the accuracy of the proposed BVS algorithm. These results suggest that the BVS framework that incorporates both the TFBS and PPI data performed better than those which incorporate no prior information (*p* = 0.99 × 10^−6^), only TFBS information (*p* = 2.05 × 10^−4^) as prior knowledge, and the sparse prior (*p* = 2.4 × 10^−6^). These results support our hypothesis that TFBS and PPI data can be collectively more predictive of potential GRNs than TFBS data alone.

**Figure 3:**
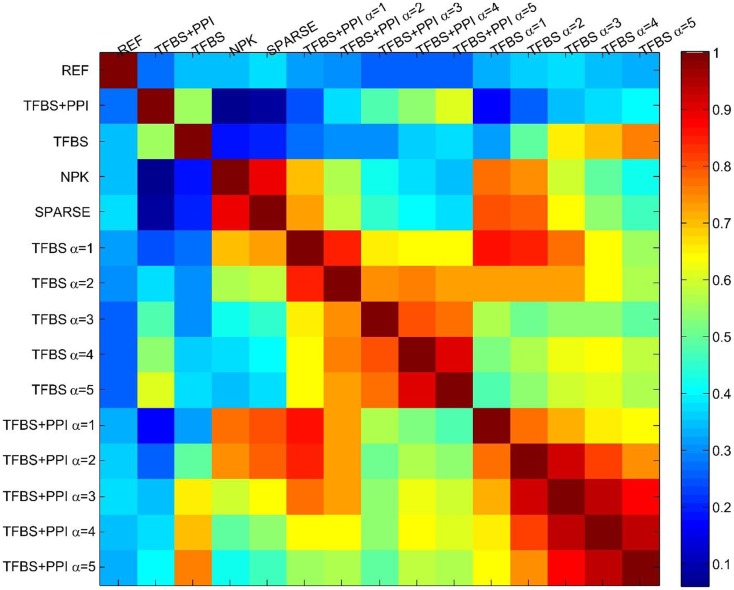
**The sensitivity of the BVS framework to the confidence parameter (α_c_)**. Here, REF represents the reference/gold standard network. TFBS represents the prior network that uses only TFBS information. TFBS + PPI represents the prior network that uses both TFBS and PPI information. No prior knowledge (NPK) represents the network that was inferred using flat prior. SPARSE represents the network that was inferred using sparse prior. TFBS α = *x* represents the posterior network inferred from **Γ**_**TFBS**_ with the confidence parameter set to α_c_ = *x*. TFBS + PPI α = *x* represents the posterior network inferred from **Γ**_**TFBS+PPI**_ with the confidence parameter set to α_c_ = *x*. The above heatmap represents the similarities (in terms of Pearson correlation coefficients) among the reference, prior, and posterior networks. Values close to 1 (dark red) represent close agreement and values close to zero (dark blue) represent a lack of agreement between network topologies. This figure suggests that the prior networks (TFBS and TFBS + PPI) do not have significant overlap with the reference network (correlation coefficients 0.42, 0.31, respectively). This is due to the fact that only 19 and 16% of the interactions that are present in the prior networks (TFBS and TFBS + PPI) are also present in the reference network (REF). Additionally, posterior networks inferred from the same prior network have a high degree of topological similarity (correlation coefficients 0.6–0.95), regardless of the value of the confidence parameter (α_c_).

Finally, I assessed the sensitivity of the BVS framework to the confidence parameter (α_c_) by looking at the agreement between results obtained under different values of this parameter. For this purpose, five different values (α_c_ = 1, 2, 3, 4, 5) of the confidence parameters were used to formulate a total of 10 prior distributions, five of these use only TFBS information and the remaining five use both TFBS and PPI information. A GRN was reconstructed using each of these prior distributions, leading to 10 inferred networks. These networks were then compared with each other to determine whether different values of the confidence parameter (α_c_) had significant effect on the network inference process. The inferred networks were then compared with the networks inferred from no prior knowledge (NPK) and sparse priors, the prior networks (**Γ**_**TFBS**_, **Γ**_**TFBS+PPI**_), and the reference network (REF). Pearson correlation coefficient was used for comparing these networks. The resulting correlation coefficients are shown in Figure 3. Values close to unity indicate high degree of similarities between networks. The networks inferred from the same type of prior distribution are in close agreement with each other, despite different values of the confidence parameter α_c_. This suggests that the proposed BVS framework is relatively insensitive to different values of α_c_. However, the networks inferred from different types of priors are mostly different from each other. Additionally, the inferred networks are also considerably different from the prior networks suggesting that the proposed Bayesian framework indeed strikes a balance between prior information and observed data.

Encouraged by the above results, I implemented the proposed BVS framework to infer the regulatory mechanisms of the human breast epithelium and compared its performance with a state-of-the-art network inference method, which relies on LASSO regression. The results of this comparison are described in detail in the next section.

## Inferring GRN of Human Breast Epithelium and Comparison with LASSO

For large-scale GRN inference, I used a set of mRNA expression measurements obtained from human epithelium at different stages of cancer development (Graham et al., 2010). The dataset was produced by Graham et al. (2010) who performed gene expression analysis of breast epithelium tissue samples obtained from 42 patients (18 cancer free, 18 had prophylactic mammoplasty, and 6 had reduction mammoplasty) in order to understand the differences in expression profiles of histologically normal breast epithelium and usual-risk controls undergoing reduction mammoplasty. These expression profiles were used to infer the GRN that governs the regulatory mechanisms of human breast epithelium. The natural genetic variations caused by SNP, copy number variations, mutation, epigenetic regulation, etc., were considered to be genetic perturbations that led to different gene expression profiles among different patients. To save computational time, only top 2000 probe sets (1337 genes) with the highest between-sample variances were selected (Table S4 in Supplementary Material). Among the selected probes, there were 93 known TFs (Table S5 in Supplementary Material) which were used as potential regulators of the selected genes for network inference.

**Figure 4:**
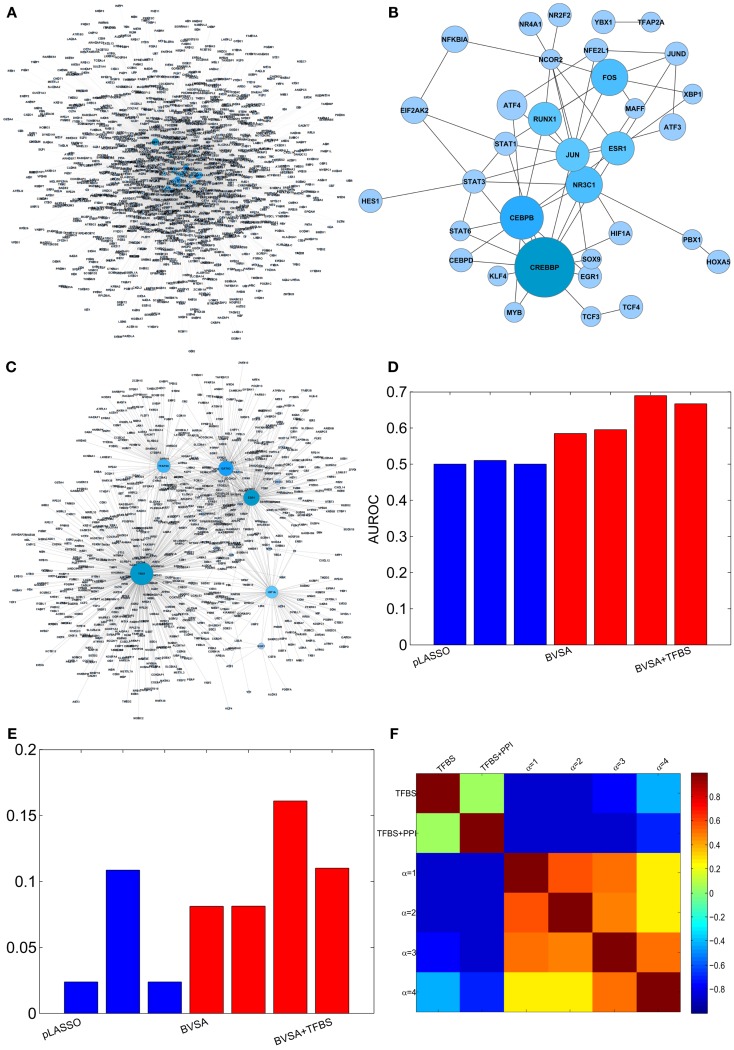
**Reconstructing GRN of human breast epithelium and comparison with LASSO**. **(A)** Prior network based on TFBS information. **(B)** PPI among TFs. **(C)** The gold standard network. **(D)** AUROCs of LASSO and BVS algorithms under different prior settings. **(E)** AUPRs of LASSO and BVS algorithms under different prior settings. **(F)** Sensitivity of the BVS algorithm to the confidence parameter (α_c_). Here, TFBS represents the prior network constructed from TFBS data, TFBS + PPI represents the prior network constructed from both TFBS and PPI information, α = 1, 2, 3, 4 represents the networks inferred from **Γ**_**TFBS+PPI**_ with confidence parameters α_c_ = 1, 2, 3, 4, respectively.

Four different prior settings were used for the BVS framework. The parameter settings for the flat and sparse priors were left the same as before. TFBS information were collected from ENCODE (Hughes et al., 2000; Ernst et al., 2010), MEME (Bailey et al., 2009), TRANSFAC (Bryne et al., 2008), and JASPER (Matys et al., 2006) to construct the prior network (**Γ**_**TFBS**_) that contains only direct gene regulations (Figure 4A). This network (**Γ**_**TFBS**_) contains 4963 number of potential gene regulations between 93 TFs and 1317 target genes (Table S6 in Supplementary Material). Information regarding PPI among TFs (Figure 4B) was collected from physical TF binding data published by Ravasi et al. (2010) (Table S7 in Supplementary Material). This information along with the TFBS data were used to construct a second prior network (**Γ**_**TFBS+PPI**_) which contains 16,372 potential regulatory interactions supported by both types of data (Table S8 in Supplementary Material). The confidence parameter (α_c_) was set to 2 and the restriction parameter (*k*) were assigned a value of 12 $\left(k=\frac{16,372}{1317}\approx 12\right)$. The above prior settings, when used with the proposed BVS framework led to four different posterior networks that were then used for performance evaluation and comparison purposes.

For performance comparison, a LASSO regression-based GRN inference algorithm (Wang et al., 2013) was selected due to recent popularity of LASSO-based methods in the network inference community (van Someren et al., 2003; Li and Yang, 2004; van Someren et al., 2006; Shimamura et al., 2007; Hecker et al., 2009, 2012; Lee et al., 2009; Charbonnier et al., 2010; Gustafsson and Hornquist, 2010; James et al., 2010; Pan et al., 2010; Peng et al., 2010; Wang et al., 2013). LASSO is a regularized version of least-square regression which uses the constraint that ||***β***||^1^, the *L*^1^-norm of the regression coefficients, is no greater than a given value. This is equivalent to an unconstrained minimization of the least-squares penalty with an added penalty λ||***β***||^1^, where λ is a constant. As the penalty is increased, LASSO regression drives more and more of the regression coefficients (***β***) to 0, leaving fewer and fewer non-zero coefficients. Both LASSO and BVS share some similarities in their core formulations but differ in some key aspects in their implementations. For instance, both these algorithms rely on linear regression models, but LASSO uses absolute shrinkage regularization to deal with curse of dimensionality where BVS uses MCMC sampling for the same purpose. Therefore, comparing the results obtained from LASSO- and BVS-based techniques may reveal the strengths and weaknesses of algorithms which rely on regularization and MCMC sampling. Similar to the BVS framework, three different prior settings were used for the LASSO-based algorithm. In the first case, no prior information was used, and in the second and third cases, **Γ**_**TFBS**_ and **Γ**_**TFBS+PPI**_ were used, respectively, as prior networks. The values of the regularization parameters were kept at their default values (λ_1_ = 0.2, λ_2_ = 0.8). This led to three different networks that were inferred by the LASSO-based algorithm.

To evaluate the accuracy of the inferred networks, I compared these to a GSN which consists of a collection of 1726 known gene regulatory interactions obtained from the HTRIdb, ConsensusPathDB and KEGG databases (Figure 4C, see Table S9 in Supplementary Material for details). The GSN contains interactions between only 27 (out of 93) TFs and their target genes. Therefore, only the regulatory activities of these 27 TFs were compared and the activities of the remaining 66 TFs were excluded from the comparison. The comparison was done using ROC and PR curves as mentioned in the previous section. The resulting AUROC and AUPR values are shown in Figures 4D,E. These results suggest that the performance of the proposed BVS algorithm increased significantly when prior information was incorporated into the inference method. In particular, TFBS and PPI data collectively were more predictive of regulatory interactions than TFBS information alone. Moreover, BVS algorithm performed better than the LASSO-based method under all circumstances. As in the previous section, the performance of BVS algorithm was found not to be sensitive (Figure 4F) to different values of the confidence parameter (α_c_).

A possible reason behind the poor performance of LASSO can be low precision of the prior networks. The prior networks used in this study have many more interactions (≈5000, 16,000) than the REF (≈1700 interactions) and therefore have very low precision. It was shown before that the performance of LASSO degrades rapidly as the precision of the prior information decreases (Wang et al., 2013). Additionally, the above results depend largely on the quality of the GSN which is a generic network consisting of the interactions involving the selected genes and TFs. This network does not necessarily reflect the tissue-specific behavior of the gene regulatory programs in breast cancer cells and therefore may not be ideal for performance evaluation purposes. However, this network was used as gold standard due to unavailability of information regarding tissue-specific GRNs.

## Discussion

In this study, I presented a new approach that incorporates TFBS data along with protein interactions among TFs in a BVS framework to infer GRNs. The main hypothesis behind this approach was that integrating protein interactions among TFs with TFBS data increases the predictive power of the inference process, especially in a variable selection setting. This was demonstrated by inferring a liver-specific transcription regulatory network and the gene regulation program of human breast epithelium, and evaluating the accuracy of the inferred networks based on known interactions. However, there are several shortcomings of the proposed data integration method. For instance, adding all indirect interactions, predicted from TF–TF PPIs, may result in a very large number of potential interactions, leading to a very low precision prior which may not contribute to the predictive power of the inference process. This issue can be mitigated by using information on protein complexes from relevant databases when these databases mature. The precision of the prior network can also be improved by removing unlikely edges that can be determined by other types of data, e.g. eQTL data.

Moreover, the proposed BVS framework relies on a linear regression model of gene regulation. Although linear regression models are extensively used by network inference community due to ease of implementation, it was recently shown that tree-based regression models may be better suitable than linear regression models in network reconstruction problems (Huynh-Thu et al., 2010). Therefore, a possible upgrade of the proposed Bayesian framework will be to replace the linear regression-based gene regulation models by tree-based regression models. Additionally, in this study, I focused mainly on two types of external data sources, consensus motif data, and PPI data. There are a plethora of other functional genomics data, e.g. GO, SNP, gene orthology, etc., which can also be predictive of potential gene regulatory interactions. Our next objective is to find a meaningful way of incorporating such information into the BVS framework.

## Conflict of Interest Statement

The author declares that the research was conducted in the absence of any commercial or financial relationships that could be construed as a potential conflict of interest.

## Supplementary Material

The Supplementary Material for this study can be found online at http://www.frontiersin.org/Journal/10.3389/fbioe.2014.00013/abstract
