# Supplementary material for: A Bayesian Framework That Integrates Heterogeneous Data for Inferring Gene Regulatory Networks
Source: Front Bioeng Biotechnol. 2014 May 20;2:13. doi: 10.3389/fbioe.2014.00013 (PMC4126456; doi:10.3389/fbioe.2014.00013)
Supplement: Supplementary file 1 [file Data_Sheet1.ZIP › SupplementaryNote1.pdf]

The Variable Selection Framework:

The expressions ($\boldsymbol{x^i}$) of a gene g^i are assumed to be linearly dependent on those ($\boldsymbol{X^i}$) of its regulators ($\boldsymbol{g^i}$):

$$x_{ij} = \sum_{k=1,k\neq i}^{k=n} A_{ik}\beta_{ik}X_{kj} + \epsilon_{ij} \qquad (1)$$

Here, $\boldsymbol{A^i} = \{A_{ik}, k = 1 \dots n\}$ is a binary vector whose non-zero elements represent potential regulators of g_i, $\boldsymbol{\beta^i} = \{\beta_{ik}, k = 1 \dots n\}$ represents the interaction strengths between g_i and its regulators and ϵ_{ik} is the error variable. The prior distributions of $\boldsymbol{A^i}, \boldsymbol{\beta^i}$ and ϵ_{ik} are shown below.

$$P(\epsilon_{ik}|\sigma^2) = N(0, \sigma^2)$$

$$P(\boldsymbol{\beta^i}|\boldsymbol{A^i}, \sigma^2, c) = N\left(0, c\sigma^2\left(\boldsymbol{X^i}^{\boldsymbol{T}}\boldsymbol{X^i}\right)^{-1}\right)$$

$$P(\boldsymbol{A^i}|\alpha_c, \boldsymbol{\Gamma^i}) \propto \exp(\boldsymbol{\Gamma^i}^T\boldsymbol{A^i}) \qquad (2)$$

Here, σ^2 is the error variance, c is the Zellner's constant which is assigned a value of $c = \max(n_p, n_i^2)$ (see the main text) where n_i is the number of non-zero elements in $\boldsymbol{A^i}$, α_c is the confidence parameter which is assigned a value of 2, $\boldsymbol{\Gamma^i}$ is the ith row of the prior network which is constructed from TFBS and PPI data. The hyper-parameter σ^2 is assumed to have Jeffry's Prior, i.e.

$$P(\sigma^2) \propto \frac{1}{\sigma^2} \qquad (3)$$

The conditional dependence of different dependent and independent variables of the BVS algorithm discussed in this paper is graphically depicted in Fig. S1.

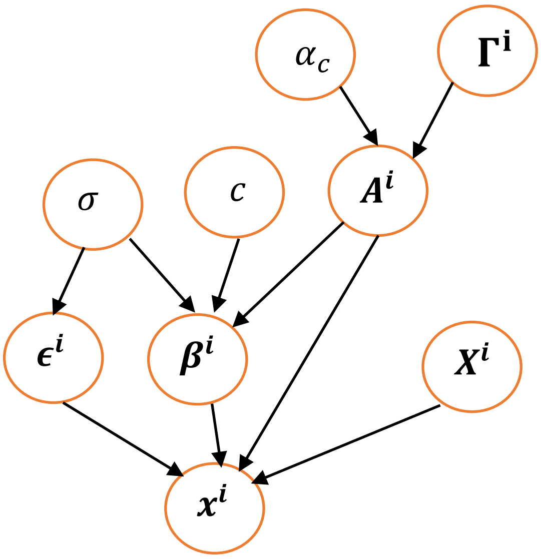

Figure S1: Conditional dependence of the variables of the BVS framework.

Based on the above priors and hyper-priors, the likelihood can be calculated as follows:

$$P(\boldsymbol{x^i}|\boldsymbol{X^i},\boldsymbol{A^i},\boldsymbol{\beta^i},\sigma^2)=\prod_{j=1}^{n_p} N\left(\sum_{k=1,k\neq i}^{k=n} A_{ik}\beta_{ik}X_{kj}\,,\sigma^2\right) \qquad (4)$$

Applying Bayes theorem:

$$P\left(A^i|\boldsymbol{X},\boldsymbol{\beta^i},\sigma^2\right)=\frac{P(\boldsymbol{x^i}|\boldsymbol{X^i},\boldsymbol{A^i},\boldsymbol{\beta^i},\sigma^2)P(\boldsymbol{\beta^i}|\boldsymbol{A^i},\sigma^2)P(\boldsymbol{A^i})P(\sigma^2)}{\sum_{\boldsymbol{A^i}} P(\boldsymbol{x^i}|\boldsymbol{X^i},\boldsymbol{A^i},\boldsymbol{\beta^i},\sigma^2)P(\boldsymbol{\beta^i}|\boldsymbol{A^i},\sigma^2)P(\boldsymbol{A^i})P(\sigma^2)}$$

$$\propto P(\boldsymbol{x^i}|\boldsymbol{X^i},\boldsymbol{A^i},\boldsymbol{\beta^i},\sigma^2)P(\boldsymbol{\beta^i}|\boldsymbol{A^i},\sigma^2)P(\boldsymbol{A^i})P(\sigma^2)$$

$$=\prod_{j=1}^{n_p} N\left(\sum_{k=1,k\neq i}^{k=n} A_{ik}\beta_{ik}X_{kj}\,,\sigma^2\right)N\left(0,c\sigma^2\left(\boldsymbol{X^i}^{\boldsymbol{T}}\boldsymbol{X^i}\right)^{-1}\right)\exp(\boldsymbol{\Gamma^i}^T\boldsymbol{A^i})\frac{1}{\sigma^2} \qquad (5)$$

Here, $P\left(A^i|\boldsymbol{X},\boldsymbol{\beta^i},\sigma^2\right)$ is the posterior distribution of $\boldsymbol{A^i}$**.** Marginalizing with respect to $\boldsymbol{\beta^i}$ and σ^2 we obtain:

$$P\left(A^i|\boldsymbol{X}\right)$$

$$\propto \iint \prod_{j=1}^{n_p} N\left(\sum_{k=1,k\neq i}^{k=n} A_{ik}\beta_{ik}X_{kj}\,,\sigma^2\right)N\left(0,c\sigma^2\left(\boldsymbol{X^i}^{\boldsymbol{T}}\boldsymbol{X^i}\right)^{-1}\right)\exp(\boldsymbol{\Gamma^i}^T\boldsymbol{A^i})\frac{1}{\sigma^2}\,d\boldsymbol{\beta^i}\,d\sigma^2$$

$$=\frac{\frac{\Gamma\left(\frac{n-1}{2}\right)}{\pi^{\frac{n-1}{2}}n^{\frac{1}{2}}}\left|\left|\boldsymbol{x^i}-\overline{\boldsymbol{x^i}}\right|\right|^{-(n-1)}(1+c)^{\frac{n-1-n_i}{2}}}{[1+c(1-R^2)]^{\frac{n-1}{2}}}\exp(\boldsymbol{\Gamma^i}^T\boldsymbol{A^i}) \qquad (6)$$

Here, $R^2=1-\frac{\left(\boldsymbol{x^i}-\boldsymbol{X^i}^{\boldsymbol{T}}\widehat{\boldsymbol{\beta^i}}\right)^T\left(\boldsymbol{x^i}-\boldsymbol{X^i}^{\boldsymbol{T}}\widehat{\boldsymbol{\beta^i}}\right)}{\left(\boldsymbol{x^i}-\overline{\boldsymbol{x^i}}\right)^T\left(\boldsymbol{x^i}-\overline{\boldsymbol{x^i}}\right)}$ is the coefficient of determination of the linear model shown in eq. 2 where $\widehat{\boldsymbol{\beta^i}}=\left(\boldsymbol{X^i}^{\boldsymbol{T}}\boldsymbol{X^i}\right)^{-\mathbf{1}}\boldsymbol{X^i}^{\boldsymbol{T}}\boldsymbol{x^i}$ is the least square estimate of $\boldsymbol{\beta^i}$, $\overline{\boldsymbol{x^i}}$ is the sample average of $\boldsymbol{x^i}$ and n_i is the number of 1s in the $\boldsymbol{A^i}$**.** The above expression can be further simplified as follows:

$$P(\boldsymbol{A^i}|\boldsymbol{X})=\frac{P(\boldsymbol{A^i}|\boldsymbol{X})P(\boldsymbol{A_0}|\boldsymbol{X})}{P(\boldsymbol{A_0}|\boldsymbol{X})}\propto\frac{P(\boldsymbol{A^i}|\boldsymbol{X})}{P(\boldsymbol{A_0}|\boldsymbol{X})}=BF(\boldsymbol{A^i}|\boldsymbol{A^0})$$

$$=(1+c)^{\frac{n-1-n_i}{2}}[1+c(1-R^2)]^{-\frac{n-1}{2}}\exp(\boldsymbol{\Gamma^i}^T\boldsymbol{A^i})$$

$$=\left[(1+c)^{-\left(\frac{n_i+1}{2}\right)}\left(1-\frac{c}{1+c}R^2\right)^{-(n_p-1)/2}\right]\exp(\boldsymbol{\Gamma^i}^T\boldsymbol{A^i}) \qquad (7)$$

Here, $\boldsymbol{A_0}$ is the null (all elements are zero) model, $P(\boldsymbol{A_0}|\boldsymbol{X})$ is the marginal posterior of the null model and can be calculated from eq. 6. by setting $R^2=0, n_i=0$. $BF(\boldsymbol{A^i}|\boldsymbol{A^0})$ is the Bayes Factor with respect to null model.
