# Supplementary figures and images for: A Bayesian Framework That Integrates Heterogeneous Data for Inferring Gene Regulatory Networks
Source: Front Bioeng Biotechnol. 2014 May 20;2:13. doi: 10.3389/fbioe.2014.00013 (PMC4126456; doi:10.3389/fbioe.2014.00013)

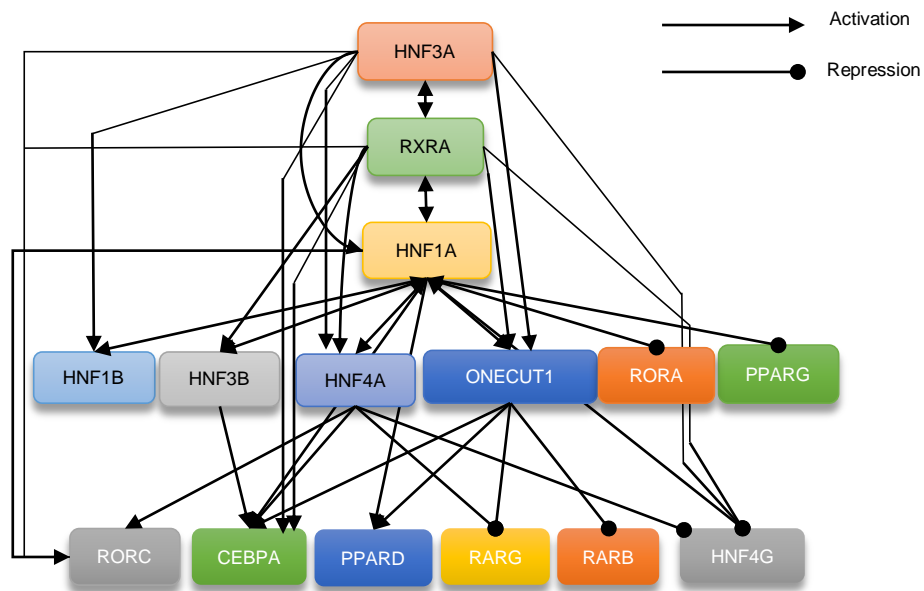

**Supplementary Figure 1:** The GSN for Tomaru et. al's dataset.

Supplement: Supplementary file 1 [file Data_Sheet1.ZIP › Supplementary Figure 1.pdf]
